# Supplementary material for: Effectiveness of Physical and Therapy Interventions for Non-ICU Hospitalized Pneumonia Patients: A Systematic Review of Randomized Controlled Trials
Source: Healthcare (Basel). 2025 Jun 16;13(12):1444. doi: 10.3390/healthcare13121444 (PMC12193386; doi:10.3390/healthcare13121444)
Supplement: Supplementary file 1 [file healthcare-13-01444-s001.zip › healthcare-3683282-supplementary.pdf]

Supplementary file. Search strategy.

Database	Search strategy
PubMed	(“Community acquired pneumonia” OR “CAP” OR “Community-acquired pneumonia”) AND ("manual therapy"[MeSH Terms]) OR (massage[MeSH Terms])) OR (exercise[MeSH Terms])) OR (stretching[MeSH Terms])) OR (physiotherapy[MeSH Terms])) OR (physical and rehabilitation medicine[MeSH Terms])) OR (gait[MeSH Terms])) OR (stability training[MeSH Terms])) OR (muscle training[MeSH Terms])) OR (strength training[MeSH Terms])) OR (resistance training[MeSH Terms])) OR (aerobic training[MeSH Terms])) OR (locomotion[MeSH Terms])) OR (weight lifting[MeSH Terms]))
Scopus	("Pneumonia" OR "community-acquired pneumonia" OR "CAP" OR "community acquired infections") AND ("manual therapy" OR "massage" OR "exercise" OR "stretching" OR "physiotherapy" OR "physical and rehabilitation medicine" OR "gait" OR "stability training" OR "muscle training" OR "strength training" OR "resistance training" OR "aerobic training" OR "locomotion" OR "weight lifting" OR "rehabilitation" OR "physical therapy modalities" OR "physical therapy")
Web of Science	("Pneumonia" OR "community-acquired pneumonia" OR "CAP" OR "community acquired infections") AND ("manual therapy" OR "massage" OR "exercise" OR "stretching" OR "physiotherapy" OR "physical and rehabilitation medicine" OR "gait" OR "stability training" OR "muscle training" OR "strength training" OR "resistance training" OR "aerobic training" OR "locomotion" OR "weight lifting" OR "rehabilitation" OR "physical therapy modalities" OR "physical therapy")
Cochrane Library	("Pneumonia" OR "community-acquired pneumonia" OR "CAP" OR "community acquired infections") AND ("manual therapy" OR "massage" OR "exercise" OR "stretching" OR "physiotherapy" OR "physical and rehabilitation medicine" OR "gait" OR "stability training" OR "muscle training" OR "strength training" OR "resistance training" OR "aerobic training" OR "locomotion" OR "weight lifting" OR "rehabilitation" OR "physical therapy modalities" OR "physical therapy")
